# Foodborne Botulism in Canada, 1985–2005

**DOI:** 10.3201/eid1906.120873

**Published:** 2013-06

**Authors:** Daniel Leclair, Joe Fung, Judith L. Isaac-Renton, Jean-Francois Proulx, Jennifer May-Hadford, Andrea Ellis, Edie Ashton, Sadjia Bekal, Jeffrey M. Farber, Burke Blanchfield, John W. Austin

**Affiliations:** Health Canada, Ottawa, Ontario, Canada (D. Leclair, J.W. Austin, J.M. Farber, B. Blanchfield);; British Columbia Provincial Health Services Authority, Vancouver, British Columbia, Canada (J.L. Isaac-Renton, J. Fung);; Nunavik Regional Board of Health and Social Services, Kuujjuaq, Quebec, Canada (J.-F. Proulx);; Public Health Agency of Canada, Guelph, Ontario (J. May-Hadford); Laboratoire de santé publique du Québec, Sainte-Anne-de-Bellevue, Quebec, Canada (S. Bekal);; ProvLab, Edmonton, Alberta, Canada (E. Ashton);; Canadian Food Inspection Agency, Ottawa (A. Ellis)

**Keywords:** botulism, Clostridium botulinum, foodborne infections, Canada, botulism, type E botulinum toxin, outbreak, serotype, antitoxin

## Abstract

During 1985–2005, a total of 91 laboratory-confirmed outbreaks of foodborne botulism occurred in Canada; these outbreaks involved 205 cases and 11 deaths. Of the outbreaks, 75 (86.2%) were caused by *Clostridium botulinum* type E, followed by types A (7, 8.1%) and B (5, 5.7%). Approximately 85% of the outbreaks occurred in Alaska Native communities, particularly the Inuit of Nunavik in northern Quebec and the First Nations population of the Pacific coast of British Columbia. These populations were predominantly exposed to type E botulinum toxin through the consumption of traditionally prepared marine mammal and fish products. Two botulism outbreaks were attributed to commercial ready-to-eat meat products and 3 to foods served in restaurants; several cases were attributed to non-Native home-prepared foods. Three affected pregnant women delivered healthy infants. Improvements in botulism case identification and early treatment have resulted in a reduction in the case-fatality rate in Canada.

Foodborne botulism, a notifiable disease in Canada, results from the ingestion of foods contaminated with preformed botulinum neurotoxin types A, B, E, or F, produced by *Clostridium botulinum* groups I and II (*1*). More rarely, outbreaks of foodborne botulism in the United States, India, and China have been caused by neurotoxigenic *C. butyricum* type E (*2**,**3*) and *C. baratii* type F (*4*). In Canada, *C. botulinum* type E has been the most common serotype since the first type E outbreak in 1944 in Nanaimo, British Columbia (reported in 1947) (*5**–**7*).

Six forms of botulism have been described in the literature (*8*), but only foodborne and infant botulism and rare cases of adult colonization have been reported in Canada (*1**,**9*). Regardless of the form or serotypes involved, however, human botulism is a medical emergency that requires rapid intervention. Because prompt administration of antitoxin can reduce the severity of the disease (*10*), the decision for treatment is based on clinical diagnosis and epidemiologic information, without laboratory confirmation.

Investigation of foodborne botulism incidents provides useful information regarding implicated foods and conditions resulting in toxin formation. The last epidemiologic review on foodborne botulism in Canada was done for the 1971–1984 period (*7*). Since then, annual summaries of botulism cases were inconsistently published through disease surveillance reports (e.g., *11**,**12*). Here, we summarize reports of all laboratory-confirmed cases of foodborne botulism in Canada during 1985–2005.

## Materials and Methods

### Data Sources

Two independent laboratory databases, maintained by the Botulism Reference Service at Health Canada, Ottawa, Ontario, and the British Columbia Public Health Reference Microbiology Laboratory, Vancouver, British Columbia, were examined for cases of foodborne botulism confirmed during 1985–2005. Information regarding the number of clinical cases, age and sex of patients, implicated food, case history, laboratory analysis, date, and location of the outbreak were extracted.

To ensure consistency in data recording and analysis throughout the study period, we used the 2009 national case definition for confirmed cases of foodborne botulism (*13*). A case of foodborne botulism is confirmed on the basis of clinical evidence and a positive laboratory specimen (i.e., detection of botulinum neurotoxin in serum, feces, gastric aspirate, or food or isolation of *C. botulinum* from feces or gastric aspirate). In addition to cases with laboratory confirmation, persons with botulism who were epidemiologically linked to a laboratory-confirmed case were considered to have confirmed, and thus reportable, cases. Detection of botulinum neurotoxin and isolation of viable *C. botulinum* from foods and clinical specimens were performed according to Health Canada method MFHPB-16 (*14*). Data on length of hospitalization were retrieved from the Hospital Morbidity Database (HMDB) of the Canadian Institute for Health Information (www.cihi.ca); all records that listed botulism in the first 3 suspected diagnostic codes were extracted for the years 1994–2005 (all years currently available). These records were then matched to the laboratory records by age, sex, date of admission, date of sample, and province of residence. Only cases with laboratory confirmation were included in the analysis of the HMDB data.

### Data Analysis

To provide a descriptive epidemiology of outbreaks of foodborne botulism in Canada during 1985–2005, we analyzed data from laboratory-confirmed outbreaks (in Canada, because of the urgency of the disease, 1 case of botulism constitutes an outbreak) in terms of case numbers and rates, demographic characteristics of patients, length of hospitalization, food types, outbreak settings, serotype, and circumstances of occurrence. The rates of disease were calculated by using census data from Statistics Canada and other sources (*15**,**16*). Statistical analysis of the HMDB data was done in SPSS version 19 (IBM, Armonk, NY, USA). The Mann-Whitney test was done to compare central tendency of the data; relationships were considered significant at p**<**0.05.

## Results

### Demographics and Incidence

**Figure 1:**
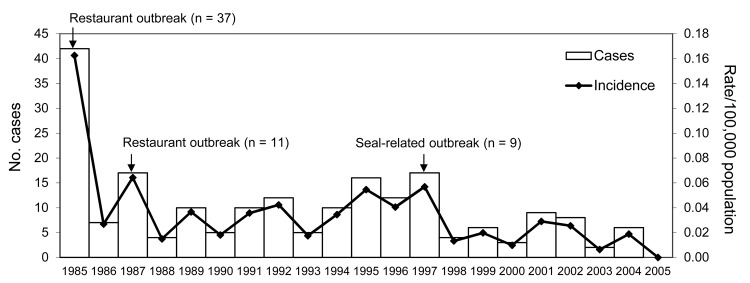
Number of cases of foodborne botulism and disease incidence (rate/100,000 population), Canada, 1985–2005.

Of 91 laboratory-confirmed outbreaks (a total of 205 cases), *C. botulinum* type E was implicated in 75 (86.2%), followed by types A (7, 8.1%) and B (5, 5.7%). Median patient age was 45 years (range 3–80 years); 93 (48.4%) were male. Three cases in the Nunavik region were recorded as repeated episodes of type E botulism, with the second episodes occurring 10–20 years after the initial intoxication. Overall, the number of outbreaks of foodborne botulism did not decrease during the study period, with a mean of 4.3 outbreaks/year. Outbreaks involved 1–37 cases, and 78% of outbreaks involved 1 or 2 cases. Mean annual incidence was 0.03 cases/100,000 population. The annual number of cases was marked by peaks associated with large outbreaks (Figure 1). The number of cases appeared to decrease during the last 5 years of the study period; lower numbers of outbreaks with multiple cases were reported (Table 1).

**Table 1:** Foodborne botulism outbreaks, cases, deaths, and case-fatality rates, by 5-year intervals, Canada, 1985–2005

Period	No. outbreaks	No. cases	No. deaths	Case-fatality rate
1985–1989	20	90	7	8.8
1990–1994	22	42	1	2.4
1995–1999	27	55	2	3.6
2000–2005	22	28	1	3.4
1985–2005	91	205	11	5.4

### Morbidity and Mortality

A total of 11 deaths were reported (case-fatality rate 5.4%). Within the 21-year study period, the case-fatality rate declined from 8.8% in 1985–1989 to 2.4% in 1990–1994 and then remained below 3.6% during the remaining 5-year intervals (Table 1). However, because mortality data were derived from the laboratory databases that do not officially keep mortality records, these data should be interpreted with caution.

Three of the reported cases occurred in pregnant women. Case history revealed that 2 of these women were exposed to type E botulinum neurotoxin and 1 to type B botulinum neurotoxin. No apparent effect on pregnancy was reported.

Severity of illness was assessed by using HMDB data for 1994–2005. The length of hospitalization was obtained for 70 patients with type E botulism (Table 2); the low number of type A and type B botulism cases recorded in HMDB did not enable valid data reporting. Although HMDB does not list deaths, the authors are aware of 1 fatal type E case from among these patients. Of botulism type E case-patients with known length of hospitalization, 37 (52.9%) were female and 33 male, which was not significantly different (p = 0.633). The median length of hospitalization was 7 days for female (mean 14.8 days) and male (mean 8.7 days) patients; these findings were not significantly different (p>0.05). The mean for the female group was influenced by 1 fatal case in which the patient was hospitalized for 225 days. When type E case-patients were subdivided based on age, age groups differed in length of hospitalization, but a trend was not apparent (Table 2).

**Table 2:** Length of hospitalization for botulism type E case-patients, by age group, Canada, 1985–2005

Age group, y	No. cases	Length of hospitalization, d		
		Mean	Median	Range
<30	5	47.6	2	1–225
30–39	8	4.6	4	1–8
40–49	18	10.8	9.5	3–29
50–59	23	6.0	4	1–18
60–69	10	15.3	15.5	3–30
>70	6	12.0	6.5	2–37
All	70	11.9	7	1–225

Hospital procedure codes were extracted from HMDB for 54 patients with type E botulism. These 54 cases represent only a subset of the total type E cases, and all were laboratory confirmed and matched with laboratory records. Intubation alone was recorded for 16 (30%) patients, antitoxin alone for 21 (39%) patients, and both for 5 (9%) patients; 12 (22%) patients received neither antitoxin nor ventilation.

For the group comprising all patients that received antitoxin (n = 26), the median length of hospital stay was 5 days (mean 15.1 days, SD 43.2), significantly (p = 0.003) shorter than for the group that did not receive antitoxin (n = 28), which had a median length of hospital stay of 11 days (mean 13.3 days, SD 8.32). For the group comprising all patients that were intubated (n = 21), the median length of hospital stay was 13 days (mean 24.5 days, SD 46.6), significantly (p<0.001) longer than for the group that did not undergo intubation (n = 33), which had a median length of hospital stay of 5 days (mean 7.58 days, SD 6.24). Intubation is likely a marker of severity of symptoms and is not thought to be the cause of increased length of hospitalization. No determination for the elapsed time period between symptom onset and the administration of antitoxin or ventilation was available.

### Geographic Distribution

**Figure 2:**
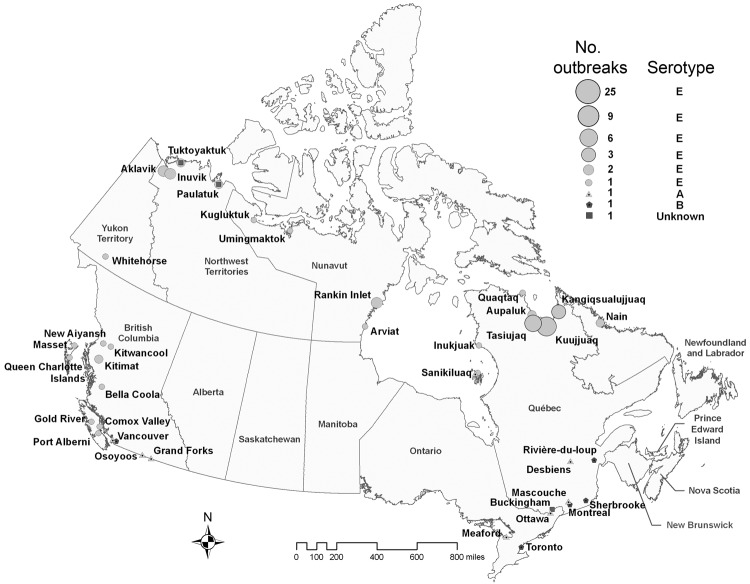
Distribution of outbreaks of foodborne botulism by serotype, Canada, 1985–2005. Circles represent type E outbreaks, triangles type A outbreaks, pentagons type B outbreaks, and squares outbreaks of unknown serotype. Circle sizes are proportionate to the number of outbreaks occurring in a given location.

Most (85, 93.4%) confirmed botulism outbreaks originated in Quebec, British Columbia, Nunavut, and the Northwest Territories. Reported cases predominantly occurred in Quebec and British Columbia, with 89 and 71 cases, respectively; these cases accounted for 78% of the total number of cases (Table 3). Of 51 outbreaks in Quebec, 45 (88%) occurred in the Nunavik region of northern Quebec; 91% of these were clustered in 3 villages of southern Ungava Bay (Kuujjuaq, Kangiqsualujjuaq, and Tasiujaq), which are inhabited by an Inuit population of 2,587 (Figure 2). In British Columbia, 9 (64%) of 14 outbreaks (20 cases) occurred in First Nations communities located along the Pacific Coast. The high number of cases recorded in the province was primarily because of 2 large restaurant-associated outbreaks in Vancouver that affected 37 persons (*17*) and 11 persons (*18*). In Ontario, 3 outbreaks were recorded, with 1 affecting 3 persons and causing 1 death. No botulism cases were reported in the provinces of Alberta, Saskatchewan, Manitoba, New Brunswick, Nova Scotia, and Prince Edward Island during the study period.

**Table 3:** Foodborne botulism outbreaks, cases, and deaths, by province/territory, Canada, 1985–2005

Province/territory	No. (%) outbreaks	No. (%) cases	No. (%) deaths
Newfoundland and Labrador	2 (2.2)	3 (1.5)	1 (9.1)
Quebec*	51 (56.0)	89 (43.4)	2 (18.2)
Ontario	3 (3.3)	5 (2.4)	1 (9.1)
British Columbia	14 (15.4)	71 (34.6)	3 (27.3)
Yukon	1 (1.1)	3 (1.5)	1 (9.1)
Northwest Territories	12 (13.2)	16 (7.8)	2 (18.2)
Nunavut	8 (8.8)	18 (8.8)	1 (9.1)
Canada†	91	205	11

*Forty-five outbreaks occurred in the Nunavik region of Northern Quebec †No laboratory-confirmed cases of foodborne botulism were reported from Alberta, Saskatchewan, Manitoba, New Brunswick, Nova Scotia or Prince Edward Island.

### Traditional Native Foods

Other than a single outbreak in Nunavut caused by consumption of fermented fish heads, all type E outbreaks with known food sources in northern Canada were linked to marine mammals, including beluga whales, seals, and walruses (Table 4). Of the 41 botulism outbreaks in Nunavik, 32 (78%) were caused by food products of seal origin; aged meat (*igunaq*) and aged flippers (*utjaq*) were most frequently implicated foods in Nunavik, accounting for 15 (47%) seal-related outbreaks. Beluga whale was involved in 16 (80%) of 20 outbreaks in Nunavut and the Northwest Territories. Eleven of these outbreaks were caused by *muktuk*, which is aged pieces of skin (with fat and meat) of the beluga whale.

**Table 4:** Foodborne botulism outbreaks, cases, and deaths, by food source and year, Canada, 1985–2005

Food source and year	Implicated food source	No. outbreaks	No. cases	No. deaths	Toxin serotype
Commercial retail foods					
1995	Pâté	1	2	0	B
2000	Cooked boneless pork	1	1	0	A
Restaurant foods					
1985	Chopped garlic in oil	1	37	0	B
1987	Bottled chanterelle mushrooms	1	11	0	A
2002	Baked potato	1	1	0	A
Home-prepared foods					
1985	Home-canned mushrooms	1	1	0	B
1988	Homemade sausages	1	1	0	E
1989	Bean soup	1	1	0	A
1991	Home-canned asparagus	1	3	1	A
1993	Homemade beef and vegetable soup	1	1	0	A
1995	Marinated and smoked fish	1	3	1	E
2000	Spaghetti sauce containing home-canned sausage	1	1	0	A
2001	Fish	1	1	0	B
Native foods					
1985–2005	Marine mammal products				
	Seal meat and fat	37	70	5	E
	Walrus meat	3	9	0	E
	Beluga meat and skin*	19	31	1	E
	Meat and fat	3	3	0	E
1985–2001	Fish products				
	Salmon eggs	8	19	3	E
	Fish and fish heads	2	2	0	E
Unknown food source*					
1990–2005	Unknown	6	7	0	B, E

*Serotype was not confirmed for 2 outbreaks involving *muktuk* and for 1 outbreak with an unknown food source.

In the First Nations communities of British Columbia, 9 outbreaks of type E botulism were associated with the consumption of aged fish products. Fermented salmon eggs (stink eggs) have been the primary source of botulism, accounting for 8 of 9 (89%) outbreaks in British Columbia coastal communities. Because no carbohydrates are available in these eggs for a fermentative transformation into organic acids, the aging process involves putrefaction rather than fermentation.

### Restaurant Food

The 1985 type B outbreak involving garlic-in-oil at a Vancouver restaurant affected 37 persons; 24 were hospitalized, and none died (*17*) (Table 4). The outbreak occurred in 2 clusters, with 11 cases in the first cluster during July 28–August 2, 1985, and 26 cases in the second cluster during August 29–September 5, 1985. In this incident, garlic-in-oil used in sandwiches was kept at room temperature for 8 months. In a 1987 botulism outbreak at a hotel restaurant in Vancouver, 11 persons became ill after consuming bottled chanterelle mushrooms contaminated with type A botulinum neurotoxin (*18*). The mushrooms were grown on Vancouver Island and bottled at the hotel using an in-house heating process. One jar recovered during the investigation was found to contain 4,000 minimal lethal dose/mL of type A neurotoxin in the liquid phase.

A third outbreak associated with a restaurant occurred in Ontario in 2002 and involved a single case of type A botulism linked to a baked potato (*19*). This patient began to show gastro-intestinal and neurologic symptoms 12 hours after eating the potato, the remains of which had been discarded and was unavailable for testing. The patient was hospitalized for >6 months and released with long-term sequelae; the patient continued to experience weakness and vision problems 4 years after the incident.

### Commercial Foods

Two unrelated incidents involving commercial ready-to-eat meat products were reported in Quebec in 1995 and 2001 (Table 4). In both instances, products that were intended to be refrigerated were stored at room temperature, which enabled growth of *C. botulinum* and toxin production. In the 1995 incident, *C. botulinum* type B was isolated from fecal samples of 2 persons and from a commercial country-style pâté. In the 2001 incident, *C. botulinum* type A was isolated from a cooked boneless pork product and from fecal and gastric liquid samples from a patient. The cooked boneless pork product was recalled as a precautionary measure.

### Home-prepared Foods

Several incidents during the 21-year study period involved non-Native home-prepared foods (Table 4). Home-canned mushrooms were linked to a type B botulism incident that affected 1 person in Quebec. Home-canned asparagus was involved in 1 type A botulism incident in Ontario; 3 persons required intensive care, and 1 died. In this incident, 8 of 10 glass jars of whole-stalk asparagus showed evidence of odorous gas production during the investigation. One additional type A case was linked to the consumption of home-canned vegetable and beef soup.

A variety of noncanned home-prepared foods, including bean soup, spaghetti sauce, sausages, and fish, were also implicated in botulism outbreaks, although most of the incidents could not be laboratory confirmed because no remaining food was available. Home-smoked fish was responsible for 3 cases of type E botulism, with 1 fatality, in the Yukon in 1995. Diagnosis and treatment with antitoxin was delayed until 3 days after the onset of symptoms. Another incident of fish consumption affected 1 tourist traveling in Quebec in 2001, but the mode of preparation was not recorded. Home-prepared spaghetti sauce made with home-canned sausage caused botulism in 1 person from Quebec in 2000. Another type A case was associated with the consumption of homemade bean soup, but its preparation was not documented.

### Laboratory Findings

Of 91 total confirmed outbreaks in Canada during the study period, 77 (84.6%) were confirmed by detection of botulinum neurotoxin only or by detection of neurotoxin and isolation of *C. botulinum* from laboratory specimens. The remaining 14 outbreaks were confirmed by detection of *C. botulinum* in gastric contents and/or fecal samples only, without detection of neurotoxin in clinical samples. Food was recovered in 69 (75.8%) of the outbreak investigations. The detection rate for botulinum neurotoxin in foods (78.3%) was found to be higher than for serum (34.9%), feces (34.5%), or gastric contents (11.0%) (Table 5). *C. botulinum* was also more frequently detected in foods (83.3%), followed by gastric contents (51.3%) and feces (40.3%). Two botulism cases were confirmed by the detection of *C. botulinum* in liver tissue during autopsies.

**Table 5:** Results of laboratory analyses of clinical and food specimens submitted for botulism investigation, Canada, 1985–2005

Specimen type	Test type	No. outbreaks	No. specimens	No. (%) specimens with positive results
Serum	BoNT	82	212	74 (34.9)
Gastric contents	BoNT	48	73	8 (11.0)
	*Clostridium botulinum*	47	78	40 (51.3)
Feces	BoNT	57	84	29 (34.5)
	*C. botulinum*	60	139	56 (40.3)
Liver				
Blood	BoNT	1	2	0 (0.0)
Tissue	*C. botulinum*	1	2	2 (100)
Cerebrospinal fluid	BoNT	1	1	0 (0.0)
Food	BoNT	69	69	54 (78.3)
	*C. botulinum*	66	66	55 (83.3)

*BoNT, botulinum neurotoxin.

## Discussion

The annual mean of 4.3 confirmed botulism outbreaks during 1985–2005 was identical to that reported for the 1971–1984 period (*7*); however, the case-fatality rate dramatically decreased during 1985–2005, from 17% to 5.4%. This decrease could reflect the increased awareness of symptoms in high-risk communities, which leads to prompt medical attention and the administration of antitoxin. Similar to 1971–1984, most cases occurred in Quebec, British Columbia, Nunavut, and the Northwest Territories, which is likely a reflection of the rate of consumption of higher-risk traditional foods in these provinces and territories. The annual mean number of outbreaks for British Columbia decreased from 0.9 to 0.7, whereas the rate for Quebec remained unchanged at 2.4 outbreaks per year. The Northwest Territories, Nunavut, and Yukon combined had 21 outbreaks during the study period, an annual average of 1 outbreak per year, unchanged from 1971–84 (*7*).

Botulism remains a public health challenge in many communities where Native foods are persistently incriminated (*7*). The mean incidence rate for all of Canada is low and is similar to that of the United States (*20*), but the rate among the Native population of Nunavik, where type E botulism linked to aged marine mammal products is endemic, is >1,600 times higher than for the rest of Canada (0.03/100,000 population for Canada vs. 50.5/100,000 population for Nunavik). Outbreaks of botulism associated with Native foods in Canada account for 83.5% of all outbreaks; in the United States, 36.3% of all outbreaks were associated with Native foods in Alaska (*20*).

Previous epidemiologic studies have identified the food types and the modes of preservation of foods frequently associated with type E botulism in northern Canada (*6**,**7*). Aged marine mammals and fish were the predominant vehicles of type E botulism before 1985 and remain responsible for most type E botulism cases in Canada. The risk for contamination of marine mammal meat during the butchering process performed under field conditions is high because of the ubiquitous presence of the organism in the coastal environment (*21**,**22*). Measures to minimize field contamination of marine mammal tissues with type E spores during the butchering and preparation of meat, fat, or skin have recently been developed (*23*). In collaboration with regional health authorities, studies directed at controlling the growth of *C. botulinum* type E in aged marine mammal products have indicated that storage temperatures <3°C will prevent toxin production during the aging process of Native foods (*23**,**24*).

Food service establishments are potential risk settings where widespread public exposure to contaminated foods may occur. The garlic-in-oil incident in 1985 was the first reported Canadian botulism outbreak associated with a food service establishment and was the largest ever reported botulism outbreak in Canada. This incident, along with a similar incident that occurred in the United States in 1989, led to regulatory changes requiring inclusion of acidifying agents in commercial garlic-in-oil products (*25*). The restaurant outbreak involving bottled chanterelle mushrooms in 1987 led the Canadian Restaurant and Foodservices Association to modify its sanitation code to recommend that in-house canned or bottled foods not be served in restaurants.

The number of botulism incidents associated with foods sold at retail remained low, with only 2 outbreaks causing illness to 3 persons. Non-Native home-prepared foods were implicated in 8 outbreaks in Canada; 2 of these were caused by home-canned vegetables. Continuous education is needed to inform consumers of the potential risks of botulism from eating home-prepared foods and to promote the use of a pressure canner according to manufacturer’s instructions and proper food storage temperatures in the home.

In addition to the classic diagnostic samples (i.e., serum, gastric content, feces, and suspect foods), the confirmation of 2 cases in 1985 was completed with the detection of viable *C. botulinum* in liver tissue during postmortem examination. Detection of viable *C. botulinum* in postmortem liver samples has been reported previously (*7**,**26*).

We found that 3 pregnant women who were diagnosed with botulism had no reported pregnancy complications. One of the patients had persistent toxemia for >10 days during the first trimester of pregnancy and later delivered a normal infant. Five previous cases of botulism during pregnancy have been reported; none of the infants appeared to have clinical botulism at birth (*27**–**31*). No evidence shows that botulinum toxin can cross the placenta (*32*) or that the neuromuscular effects of botulinum toxin on the mother would affect the development of the fetus (*28*).

Type A botulism is recognized to be more severe than type E botulism because of the higher proportion of patients who require intubation (*33*). Hughes et al. reported the mean length of hospitalization as 63 days for type A and 21 days for type B botulism (*34*), longer than the 11.9 days determined in this study for type E botulism. Administration of antitoxin has been shown to reduce risk for death and shorten the course of type A botulism (*10*). The data in this study showed that antitoxin shortened the median length of hospitalization for patients with type E botulism from 11 days to 5 days.

The overall case-fatality rate from botulism in Canada has decreased from 17.2% to 5.4%, approaching the rate in United States (*20*), which can likely be attributed to the early recognition and medical management of type E botulism cases in First Nations and Inuit communities. Most foodborne botulism outbreaks in Canada continue to occur among First Nations and Inuit people, a trend that has not changed since a 1974 comprehensive review of botulism cases, which attributed most cases of type E botulism to aged marine mammal and fish products (*6*). However, extensive progress has been made in improving case identification and early treatment, which has led to a substantial decrease in the case-fatality rate.
